# CRABP1 Signalosomes in Cellular Stress Response and Health Maintenance

**DOI:** 10.33696/signaling.6.141

**Published:** 2025

**Authors:** Jennifer Nhieu, Fatimah Najjar, Li-Na Wei

**Affiliations:** 1 Department of Pharmacology, University of Minnesota Medical School, Minneapolis, MN 55455, USA

**Keywords:** Cell signaling pathways, Molecular signaling, Stress response

## Introduction

CRABP1 is an evolutionarily conserved retinoic acid (RA) binding protein that was originally characterized to bind and sequester cytosolic RA [1,2]. Classical RA signaling involves RA binding to nuclear retinoic acid receptors (RARs) to regulate gene transcription [3]. However, recent studies have established that CRABP1 in fact forms protein complexes, with or without RA, in the cytoplasm to modulate (mostly suppress) specific signaling pathways in a cell context-dependent manner. These CRABP1-complexes are named “CRABP1-signalosomes”. RA binding to CRABP1 (holo-CRABP1) is known to enhance the formation of these signalosomes, and therefore, RA can enhance the modulatory effects of CRABP1 signalosomes [1,4–6]. As such, CRABP1 signalosomes provide a novel mechanism to modulate specific cell signaling pathways to affect cellular health and functions even without RA. Whereas in the presence of RA, the modulatory (suppressive) effects of CRABP1 signalosomes are enhanced in a RA dose-dependent manner. Molecular, cellular, and structural studies validated the existence of at least two CRABP1-signalosomes that modulate the health of various organ systems, which are the CRABP1-mitogen activated protein kinase (MAPK) signalosome [5] and CRABP1-Ca^2+^/calmodulin-dependent kinase II (CaMKII) signalosome [7]. The physiological roles of these two CRABP1 signalosomes were revealed in studies of the *Crabp1* knockout (CKO) mouse model, including modulating neural stem cell pools [8], cardiac protection from beta-adrenergic insult [6,9], protection from high fat diet induced obesity [10], regulating exosome [11,12] and adipokine secretion [13], maintaining motor neuron (MN) [14] and thyrocyte health [15], and modulating MN’s stress response [16]. As such, we propose CRABP1 signalosomes as important players in maintaining systemic homeostasis and reducing disease vulnerability. Here, we discuss and speculate on additional CRABP1 signalosomes identified recently, particularly those pertinent to cellular stress responses. Additionally, from our recent exploitation of human bioinformatics [13], we highlight the valuable translational application of targeting specific CRABP1 signalosomes in disease prevention and/or developing therapeutics that modulate the cellular stress response.

The cellular stress response is a coordinated process in response to environmental stressors to protect and maintain the integrity of the cell. The accumulation of unfolded proteins in particular can induce a variety of deleterious consequences such as increased protein aggregation, organelle dysfunction, and oxidative stress, etc. [17]. The unfolded protein response (UPR) is an organelle-specific and highly conserved stress response that can occur in the endoplasmic reticulum (ER) [18] and mitochondria [19]. The ER-UPR is executed through three major pathways mediated by eukaryotic translation initiation factor 2 alpha (eIF2α), activating transcription factor-6 (ATF6), and inositol-requiring enzyme 1 alpha (IRE1α). eIF2α is activated by the upstream eIF2α kinases, protein kinase RNA-like ER kinase (PERK), general control nonderepressible 2 (GCN2), and heme-regulated inhibitor (HRI), which, in response to specific stressors, phosphorylate eIF2α to halt global translation thereby reducing protein load. ATF6 is a membrane-bound transcription factor that, upon ER stress, is transported to the Golgi for proteolytic cleavage to release its cytosolic domain, which then enters the nucleus to induce the expression of genes involved in protein folding, ER-associated degradation (ERAD), and lipid synthesis. IRE1α, the most evolutionarily conserved member, is a sensor for ER stress that acts by splicing XBP1, a transcription factor that induces the expression of various stress response genes such as chaperones to expand the functional capacity of the ER. The mitochondrial UPR (mt-UPR) response is mainly mediated by phosphorylated eIF2α which induces the translation of transcription factors C/EBP homologous protein (CHOP), activating transcription factor 4 (ATF4), and activating transcription factor 5 (ATF5) to promote mitochondrial proteostasis by enhancing molecular chaperone activity and protein degradation processes [19].

## CRABP1 Signalosomes in Stress Response

Our study showed that depleting CRABP1 in MNs caused mitochondrial defects manifested as reduced mitochondrial DNA content and impaired metabolism [16]. Furthermore, CKO MNs had defects in antioxidant signaling pathways and mt-UPR. The failure to engage mt-UPR in CKO MNs was attributed to reduced phosphorylation of eIF2α [16]. Thus, losing CRABP1 in MNs could result in a reduced capacity to engage mitochondrial stress response, and consequentially, failure to remove reactive oxygen species (ROS) and unfolded proteins. Our recent proteomic studies [11] and molecular experiments to identify protein complexes (JN, unpublished) found that eIF2α forms a protein complex with CRABP1, suggesting the existence of a CRABP1–eIF2α signalosome in MNs. Additional studies revealed CRABP1 interacting with IRE1α in thyrocytes to modulate IRE1α phosphorylation and clustering, suggesting the existence of a CRABP1-IRE1α signalosome in thyrocytes (FN, unpublished).

These observations further expand the repertoire of cell context-dependent CRABP1 signalosomes. Of particular significance is the physiological role of CRABP1 signalosomes in certain highly specialized and vulnerable cell types. For instance, MNs are most vulnerable to excitotoxic insults because of their high-demand activities which require proper and timely stress responses to maintain protein homeostasis, prevent oxidative damage, and support mitochondrial function [20]. Thyrocytes have a high demand for ER-related functions given their need for expressing large amounts of thyroglobulin, a large protein that requires extensive post-translational modifications for the synthesis of thyroid hormones [21]. Thus, thyrocytes especially require protection against unfolded protein accumulation and stressors associated with secretion [21]. Importantly, CRABP1 is highly expressed in both MNs and thyrocytes, presumably because of their needs for CRABP1-signalosomes to effectively engage their stress responses and maintain health.

## *CRABP1* Gene Expression in Human Health and Disease

The highly conserved functions of CRABP1 signalosomes are consistent with the tight regulation of *CRABP1* gene expression. The mouse *Crabp1* gene is known to be stringently regulated, which involves specific epigenetic and physiological factors [22]. Aberrant expression or depletion of *Crabp1* negatively affects cellular health and organismal fitness [23–28]. This is supported by the various disease phenotypes revealed in CKO mice, as well as several human diseases where CRABP1 expression was found to be significantly down-regulated [1]. Presumably, strict regulation of *CRABP1* gene provides a means to regulate/safe-guard the formation and “dose” of CRABP1-signalosomes. Changes (increases) in CRABP1 expression may reflect a cell’s need for stronger stress response–related activities. This could be translationally useful in a clinical setting, such as indicating the overall health of certain cell populations and tissue types. Therefore, in certain vulnerable cell populations, proper expression of *CRABP1* would serve as a unique marker of cytoprotection; whereas its depletion would indicate increased disease vulnerability.

## Translational Applications and Conclusion

In the clinic, RA is used as a highly efficacious therapeutic agent for rare leukemias and dermatological disorders [29,30]. However, RA is known to cause toxic side-effects such as teratogenicity and differentiation syndrome, both of which are mediated by RARs [31–33]. Therefore, in translational applications of RA, RAR-associated retinoid toxicities are of primary concern. Given the documented proof-of-principle that CRABP1-selective ligands can be designed to target specific signaling pathways [5,34,35], the translational value of targeting CRABP1-signalosomes cannot be ignored. To this end, CRABP1 signalosomes provide pharmacological targets in diseases such as neurodegeneration, thyroid dysfunction, heart failure, obesity and immune functions. Therefore, in translational applications, CRABP1-selective (without acting on RARs) ligands can be applied to enhance the modulatory effects of CRABP1 signalosomes without eliciting retinoid toxicity. Furthermore, CRABP1-selective ligands can be designed to target (bias) signaling toward particular pathways active only in certain cell populations, enabling modulation of only disease-relevant pathways in specific cell types of interest, thus further reducing the likelihood of toxicities. With regards to newly uncovered CRABP1-eIF2a and -IRE1a signalosomes that modulate stress response, future studies are needed to elucidate their mechanisms of action and to identify/synthesize specific ligands to exploit their translational potential, such as to enhance cellular stress response and maintain the health of various cell types.
